# Four variants in transferrin and *HFE *genes as potential markers of iron deficiency anaemia risk: an association study in menstruating women

**DOI:** 10.1186/1743-7075-8-69

**Published:** 2011-10-06

**Authors:** Ruth Blanco-Rojo, Carlos Baeza-Richer, Ana M López-Parra, Ana M Pérez-Granados, Anna Brichs, Stefania Bertoncini, Alfonso Buil, Eduardo Arroyo-Pardo, Jose M Soria, M Pilar Vaquero

**Affiliations:** 1 Department of Metabolism and Nutrition, Institute of Food Science and Technology and Nutrition (ICTAN), Spanish National Research Council (CSIC), Madrid, Spain; 2 Department of Toxicology and Health Legislation, Faculty of Medicine, Complutense University of Madrid, Spain; 3 Unit of Genomic of Complex Diseases, Institute of Biomedical Research (II-B Sant Pau), Barcelona, Spain; 4 Department of Biology, University of Pisa, Pisa, Italy

**Keywords:** Transferrin gene, *HFE *gene, serum transferrin, transferrin saturation, iron deficiency anaemia, SNP, menstruating women, iron intake, association study, genetic markers

## Abstract

**Background:**

Iron deficiency anaemia is a worldwide health problem in which environmental, physiologic and genetic factors play important roles. The associations between iron status biomarkers and single nucleotide polymorphisms (SNPs) known to be related to iron metabolism were studied in menstruating women.

**Methods:**

A group of 270 Caucasian menstruating women, a population group at risk of iron deficiency anaemia, participated in the study. Haematological and biochemical parameters were analysed and 10 selected SNPs were genotyped by minisequencing assay. The associations between genetic and biochemical data were analysed by Bayesian Model Averaging (BMA) test and decision trees. Dietary intake of a representative subgroup of these volunteers (n = 141) was assessed, and the relationship between nutrients and iron biomarkers was also determined by linear regression.

**Results:**

Four variants, two in the transferrin gene (rs3811647, rs1799852) and two in the *HFE *gene (C282Y, H63D), explain 35% of the genetic variation or heritability of serum transferrin in menstruating women. The minor allele of rs3811647 was associated with higher serum transferrin levels and lower transferrin saturation, while the minor alleles of rs1799852 and the C282Y and H63D mutations of *HFE *were associated with lower serum transferrin levels. No association between nutrient intake and iron biomarkers was found.

**Conclusions:**

In contrast to dietary intake, these four SNPs are strongly associated with serum transferrin. Carriers of the minor allele of rs3811647 present a reduction in iron transport to tissues, which might indicate higher iron deficiency anaemia risk, although the simultaneous presence of the minor allele of rs1799852 and *HFE *mutations appear to have compensatory effects. Therefore, it is suggested that these genetic variants might potentially be used as markers of iron deficiency anaemia risk.

## Introduction

Iron deficiency is one of the leading risk factors for disability and mortality worldwide, affecting both developing and developed countries with major consequences for human health as well as social and economic improvement. An estimated two billion people are affected, and menstruating women and children are populations at risk [1].

Iron deficiency anaemia is caused by a wide variety of factors that can be isolated, but more often coexist [2]. It results from any situation in which dietary iron intake does not meet the body's demands. Physiological blood loss frequently contributes to the negative iron balance, but genetic factors also play a role [3].

It is well established that in a situation of iron-deficiency the supply of iron to transferrin is compromised, increasing the serum levels of the protein while transferrin saturation and total iron binding capacity are decreased; this leads to ferritin stores being progressively diminished [2]. More recently, hepcidin has emerged as the central regulatory molecule of systemic iron homeostasis, inhibiting ferroportin which mediates iron export from hepatocytes, duodenal enterocytes and macrophages. Hepcidin mRNA is transcriptionally regulated through at least three pathways: interleukin 6 stimulates hepcidin expression through the STAT3 signaling pathway; bone morphogenetic proteins (BMP) increase hepcidin expression through the haemojuvelin/BMP receptor/Smad 4 pathway; and transferrin stimulates hepcidin expression through a transferrin receptor 2 (TfR2)/HFE mediated pathway [4].

Mutations of several genes implicated in iron-overload have been widely studied. Haemochromatosis can be caused by mutations affecting any of the proteins that limit the entry of iron into the blood. In humans, mutations in *HFE, TfR2*, haemojuvelin and ferroportin genes can result in haemochromatosis [5].

However, limited information is available about gene variants associated with iron deficiency anaemia [6]. Atransferrinemia, due to rare mutations in the transferrin gene (*TF*), leads to low or undetectable levels of the carrier protein [7]. Mutations in the divalent metal transporter 1 gene have been found in patients with microcytic anaemia, low serum ferritin, and liver iron overload [8]. Recent studies revealed that mutations in the matriptase gene (*TMPRSS6*) cause iron-refractory iron deficiency anaemia [9]. Presence of the mutation G277S of the *TF *gene alone does not affect iron absorption in iron deficient women [10-12] and it has been suggested that a combination of polymorphisms is involved in iron metabolism [13].

The development of genome-wide association studies (GWAS) make it possible to associate genetic variations, such as single nucleotides polymorphisms (SNPs), with traits that could be related to a disease. Recent GWAS studies are available on clinically relevant haematological traits that determine iron status, such us haemoglobin levels, mean corpuscular volume, serum ferritin, serum transferrin and transferrin saturation [14-17]. Benyamin et al. (2009) demonstrated that three variants in *TF *plus the *HFE *C282Y mutation explain approximately 40% of genetic variation in serum transferrin [18]. Remacha et al. (2006) detected two quantitative trait loci that suggest linkage to soluble transferrin receptor [19].

All of these studies provide insight into haematopoietic pathways and reveal genetic variants that may predispose some individuals to iron deficiency and related disorders. The present study is part of a wider project aimed at investigating the influence of dietary, physiological, and genetic risk factors on iron deficiency anaemia. In this work, the associations between iron status biomarkers and 10 SNPs known to be related with iron metabolism were studied in menstruating women, a population group at risk of iron deficiency anaemia.

## Design and methods

### Subjects

A group of 270 menstruating women participated in the study. The inclusion criteria were: Caucasian, aged 18-45 years, non-smoker, non-pregnant, non-breastfeeding. Subjects were excluded from the study if they had amenorrhea, menopause, thalassaemia, haemochromatosis, chronic gastric diseases (inflammatory bowel disease, Crohn disease, gastric ulcers, celiac disease, haemorrhagic diseases) or renal disease.

The study protocols were approved by the Clinical Research Ethics Committee of *Hospital Puerta de Hierro*, Madrid and the Spanish National Research Council (CSIC), Madrid, Spain.

### Dietary control and anthropometric measures

The dietary intake and anthropometric measures of a randomly selected subgroup of these volunteers were assessed (n = 141). They completed a 72-h detailed dietary intake report, previously validated and proved valuable to assess nutrient intake [20,21], specifying the types of food consumed and serving weights. Daily food, energy intake, nutrient intake and energy provided by macronutrients were calculated by a computer application using the Spanish Food Composition Database [22].

Anthropometric measures were taken using standardised procedures. Body weight was measured with a calibrated Seca scale (to a precision of 100 g) and height was measured at baseline with a stadiometer incorporated into the scale. Body mass index (BMI) was calculated as weight/height squared (kg/m^2^).

### Blood sampling and biochemical assays

Blood samples were collected by venipuncture after a 12-h fasting period, between 08:00 h and 09:00 h. Serum and plasma were obtained after centrifugation at 1000 g for 15 minutes and stored at -80°C.

Total red blood cells, haematocrit, mean corpuscular volume (MCV) and haemoglobin were determined following standard laboratory techniques using the Symex NE 9100 automated haematology analyser (Symex, Kobe, Japan). Serum iron, serum ferritin and serum transferrin were determined by the Modular Analytics Serum Work Area analyser (Roche, Basel, Switzerland). Transferrin saturation (%) was calculated as follows: serum iron (μmol/L)/TIBC (μmol/L) × 100, where TIBC is total iron binding capacity, calculated as 25.1 × transferrin (g/L).

### SNP's selection and genetic analysis

A total of 10 SNPs, known to be related to iron metabolism, were selected from the bibliography. Six of them (rs3811647, rs1799852, rs2280673, rs1800562, rs4820268 and rs855791) were described in independent GWAS to be related to iron biomarkers [14-18] and three more (rs16826756, rs2673289 and rs1375515) were selected by The HapMap Project (http://www.hapmap.org/) from two linkage signals described by Remacha et al [19]. The last one (rs1799945) was chosen for its importance in iron-related diseases [23].

A 10-multiplex minisequencing assay (SNaPshot) was developed to genotype the 10 SNPs. Conditions of this multiplex method were reported previously [24].

### Statistics analysis

A normal distribution of the parameters was determined by the Kolmogorov-Smirnov test. Serum ferritin values were log transformed for statistical testing. Genotype data quality was verified for each SNP by testing for Hardy-Weinberg equilibrium using Chi-squared test. Pearson's correlation was used to study the associations between quantitative variables.

Bayesian Model Averaging (BMA) was used to analyse the associations between SNPs and variables (iron status parameters), assuming a co-dominance model. BMA accounts for the model uncertainty inherent in the variable selection process by averaging over the best models according to approximate posterior model probability. BMA estimates all of the possible models and calculates a probabilistic average of the effect of each SNP. The significance threshold was a posterior effect probability (*P*(β≠0|*D*)) ≥ 75%.

The proportion of the variance of one parameter explained by one SNP is calculated as follows: [Var(fenotype)-Var(e)]/Var(fenotype), where Var(fenotype) is the variance of the parameter and Var(e) is the variance of the residuals of the best model.

The relations between SNPs and variables were also tested by a Chi-square automatic interaction detection decision tree. Data are presented in a hierarchical way according to the SNP that better explains the differences in the studied variable. Each generated subdivision is again divided according to the existence of new predictors with a significant effect. The minimum size a node could present to be divided to was 20 individuals and the minimum size in a child node was 5 individuals, and the significance threshold was p ≤ 0.01.

Chi-squared test was performed by GenePop software (version 4.0). The Kolmogorov-Smirnov test, Pearson's correlation and decision tree were carried out by SPSS statistical package for Windows (version 17.0) and the BMA analysis was carried out using R statistic package.

## Results

Age of the participating women was 24.3 ± 4.8 years (mean ± SD) and body mass index (kg/m^2^) 21.7 ± 2.2. There were no significant differences between iron status biomarkers of the total group and the subgroup of volunteers whose dietary intake was assessed (Table 1). Dietary characteristics of the subgroup are presented on Table 2. No significant associations between nutrient intake and iron status parameters were found.

**Table 1:** Iron biomarkers of the total group and the subgroup of volunteers

	Total group (n = 270)	Subgroup (n = 141)
Haemoglobin (g/dl)	13.1 ± 0.9	13.2 ± 0.9
Haematocrit (%)	39.2 ± 2.7	39.2 ± 2.8
Mean Corpuscular Volume (fl.)	87.2 ± 5.0	86.9 ± 5.0
Serum Ferritin (ng/ml)	25.1 ± 21.3	25.5 ± 16.9
Serum Transferrin (mg/dl)	307.5 ± 54.1	311.5 ± 56.7
Serum iron (μg/dl)	81.7 ± 37.5	80.0 ± 36.
Transferrin saturation (%)	19.4 ± 10.2	18.8 ± 8.7

Differences between groups were not significant. Data are mean ± SD

**Table 2:** Energy and nutrient intake

Energy (kcal)	2125 ± 578
Protein (g)	83.3 ± 22.4
Carbohydrate (g)	215.7 ± 63.7
Lipid (g)	97.4 ± 30.4
Protein (% energy)	15.8 ± 6.6
Carbohydrate (%energy)	44.1 ± 7.2
Lipid (%energy)	40.3 ± 8.0
Dietary fiber (g)	19.2 ± 7.0
Calcium (mg)	937 ± 306
Iron (mg)	14.1 ± 4.2
Vitamin C (mg)	124.0 ± 58.5

Data are mean ± SD. n = 141 volunteers

There were significant negative correlations between serum transferrin and haemoglobin, haematocrit, MCV, serum ferritin, and transferrin saturation (p < 0.001), but the correlation between serum transferrin and serum iron was not significant. All of the studied SNPs were in Hardy-Weinberg equilibrium and presented a minor allele frequency (MAF) higher than 0.01, therefore none of them were removed from the study.

Table 3 presents the results of the association analysis between the 10 SNPs and serum transferrin, obtained by BMA. It contains the posterior means (the mean effect of the SNP across all the calculated models), standard deviations and posterior effect probabilities (*P*(β≠0|*D*)) of the SNPs to be associated with serum transferrin, and shows the models with the highest posterior probabilities, from a total of 2^10 ^possible models. Four SNPs, (rs3811647, rs1800562, rs1799945, rs1799852) were included in the best model according to its posterior model probability, 43.8%, which is much higher than that of the second model (18.3%).

**Table 3:** BMA association analysis between the 10 SNPs and serum transferrin

SNP	***P*(β≠0**|***D*)**	**Mean β**|***D***	**SD β**|***D***	Model 1	Model 2	Model 3	Model 4	Model 5
Intercept	100	304.746	6.724	307.878	300.740	301.542	305.256	303.681
rs4820268	4.6	0.205	1.318					4.475
rs855791	3.5	0.109	0.987					
rs1799852	78	-15.896	10.747	-20.255		-21.078	-20.386	-20.314
rs2280673	2.8	0.039	0.812					
rs1800562	100	-44.213	12.089	-44.875	-45.083	-40.336	-44.997	-44.276
rs3811647	100	21.223	5.224	20.322	24.300	20.720	20.267	20.042
rs2673289	4.4	0.190	1.278					
rs1375515	2.8	0.037	0.810					
rs1799945	85.6	-13.482	7.486	-15.737	-16.293		-15.044	-15.134
rs16826756	5.5	0.357	1.961				6.514	

nVar				4	3	3	5	5
r2				0.169	0.146	0.142	0.173	0.172
BIC				-27.431	-25.688	-24.606	-23.278	-22.919
Posterior model probability				0.438	0.183	0.107	0.055	0.046

*P*(β≠0|D): Probability of the SNP to be associated to the variable. Mean β|*D*: Posterior mean of the beta parameter (weighted average of the posterior means of beta under each of the models). SD β|*D*: Standard deviation of each beta. Model1...5: Most probable multiple-SNP models. nVar: Number of SNPs included in the model. BIC: Bayesian Information Criterion. r2: Coefficient of determination

The SNPs rs3811647 and rs17999852, located in the transferrin gene, yielded probabilities of 100% and 78% respectively of being associated with serum transferrin. Each A allele of the rs3811647 has an additive effect of 20.32 in the levels of serum transferrin while the T allele of rs1799852 presents an reducing effect of 20.25. These two SNPs explained 8.08% (rs3811647) and 5.55% (rs1799852) of the total variation of serum transferrin.

The SNPs rs1800562 (C282Y) and rs1799945 (H63D), located in the *HFE *gene, yielded probabilities of 100% and 85.6% respectively of being associated with transferrin levels. The A allele of rs1800562 accounts for a mean reduction of 44.87 in the levels of transferrin, which is the largest effect out of the four SNPs included in the best model, whereas SNP rs1799945 presents a negative mean effect of each G allele of 15.73. These two SNPs explain 3.07% and 2.40% of the total variation in transferrin respectively.

In the best model, the total phenotypic variation of serum transferrin explained by these SNPs (rs3811647, rs1799852, rs1800562 and rs1799945) is 16.9%.

There were no associations between SNPs rs4820268, rs855791, rs2280673, rs2673289, rs137515, rs16826756 and levels of serum transferrin (Table 3). The associations between any of the SNPs and the other studied iron biomarkers were not significant by BMA (models not presented).

**Figure 1:**
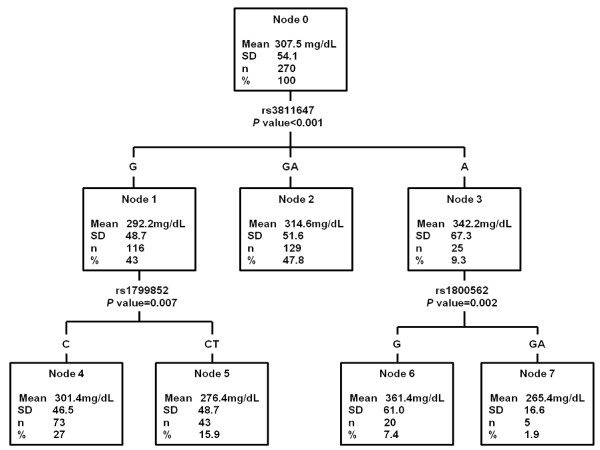
**Decision tree built with serum transferrin as dependent variable and the 10 SNPs as independent variables or factors**. %: Percentage of the total sample included in each node.

**Figure 2:**
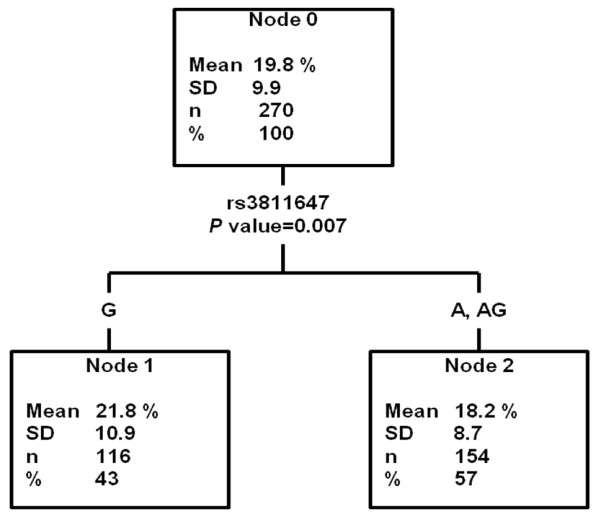
**Decision tree built with transferrin saturation as dependent variable and the 10 SNPs as independent variables or factors**. %: Percentage of the total sample included in each node.

Figures 1 and 2 show the decision trees for serum transferrin and transferrin saturation. With regard to serum transferrin, rs3811647 is the SNP that better explains this variable. Serum transferrin was significantly higher in AA women (342.2 ± 67.3 mg/dL) than AG heterozygous (314.6 ± 51.6 mg/dL) and GG homozygous (292.2 ± 48.7 mg/dL) (p < 0.001). The GG node for SNP rs3811647 is subdivided into two subgroups for the presence of rs1799852 (nodes 4 and 5). The CC genotype for this SNP presented significantly higher values of serum transferrin levels (301.4 ± 46.5 mg/dL) than the CT genotype (276.4 ± 48.7 mg/dL). The homozygous AA node for rs3811647 is subdivided into two other subgroups for the presence of SNP rs1800562 (C282Y) (nodes 6 and 7). The GG genotype for this SNP presented significantly higher values of serum transferrin levels (361.4 ± 61.0 mg/dL) than the GA genotype (265.4 ± 16.6 mg/dL). No subjects homozygous for T in rs17999852 or for A in C282Y mutation were found.

Accordingly, rs3811647 was also found to be the SNP that better explained transferrin saturation (Figure 2), with significantly lower values in AA and AG women (18.2 ± 8.7%) compared to GG homozygous women (21.8 ± 10.9%) (p = 0.007).

## Discussion

The present study shows that four SNPs, two in the *TF *gene and two others in the *HFE *gene, explain a large proportion of the genetic variation of serum transferrin in a group of menstruating women. In the best model calculated by BMA, the total phenotypic variation of serum transferrin explained by these SNPs is 16.9%. The estimated heritability of serum transferrin for Caucasian woman, which is the proportion of phenotypic variation in a population due to genetic variation, is 0.49 [25]. Therefore, these four SNPs can explain approximately 35% of the total genetic variation or heritability for serum transferrin in this population group (calculated as 16.9/0.49).

The dietary study showed that mean dietary iron intake was below the Recommended Dietary Allowance for this population group (18 mg/day) [26], which, as previously reported by our research group [27,28], is not easily reached in menstruating women. Although the influence of dietary components on iron absorption is well known [29], in the present study no association between nutrient intake and iron biomarkers was found. This is outstanding taking into account the wide range in iron biomarker levels that these volunteers presented. Moreover, these results are in agreement with other findings of our research group obtained in a study that compared iron status after consuming red meat or fish-based diets [27].

Serum transferrin and transferrin saturation are valid parameters that reflect iron status in this group of menstruating women as serum transferrin was inversely correlated with serum ferritin, haemoglobin, MCV, haematocrit and transferrin saturation [2].

Regarding the SNPs in *TF*, the two statistical analyses used led to coincident results, indicating that the presence of the minor allele A in rs3811647 is related to higher circulating transferrin levels whereas the minor allele T in rs1799852 relates to lower levels. These results from a sample of menstruating Caucasian women are consistent with and replicate data obtained by GWAS in other population groups [17,18,30].

SNP rs3811647 is situated in intron 11 and rs1799852 is a non-synonymous coding SNP in exon 17 of *TF*. As these *TF *variants are associated with serum transferrin concentration, they would appear to be involved at a transcriptional or postranscriptional level [31]. In this sense, further studies should be done in order to study the influence that these two SNPs could have on the action of these transcription factors and on transferrin expression.

Present results also show that rs3811647 is associated with transferrin saturation, in agreement with the association observed with serum transferrin and with findings obtained in other populations [18]. Considering that transferrin saturation is a marker of the iron supply to tissues, A carriers present a reduction in iron transport to tissues, as occurs in iron deficiency anaemia. Another study found that SNP rs3811647 was also associated with ferritin, although to a lesser degree than with transferrin and transferrin saturation [18]. In the present study, however, an association between this SNP and serum ferritin was not observed, which can be explained as ferritin is the final marker of iron stores, indirectly related to iron transport, and also due to the sample size.

The other two SNPs included in the best model for the BMA test, rs1800562 and rs1799945 (C282Y and H63D mutation respectively), are in *HFE*. The minor allele of C282Y was associated with lower levels of serum transferrin, which is consistent with other studies [17,18,30]. Moreover, the present study shows that H63D was also associated with serum transferrin. Both SNPs are well known to be related to type I haemochromatosis [5], however the information concerning their roles in iron deficiency is very scarce. High iron status was observed in heterozygotes women, for either C282Y or H63D, compared with women lacking these mutations [32-34]. The present results confirm previous observations of our group [13] and support the hypothesis that C282Y and H63D mutations could have a protective effect against the development of anaemia in menstruating women. In this line, studies suggest that the haemochromatosis gene may have spread because of a selective advantage through protection of heterozygotes against iron deficiency [33,35].

The study of interactions of these variants shows that the presence of G in rs3811647 and T in rs1799852 has a cumulative effect, with carriers of these variants presenting lower serum transferrin levels (Figure 1). In the presence of the minor allele A in rs3811647, that has been clearly associated with higher transferrin levels, the simultaneous presence of A in C282Y has a compensating favourable effect.

These four SNPs in *TF *and *HFE *may play a role in iron regulation because, as suggested in a recent study, the Tf/TfR2/HFE complex is critical for hepcidin regulation, and the concentration of diferric serum transferrin may act as a positive regulator, increasing hepcidin expression [36]. Mutations in either *TfR2 *or *HFE *can result in iron overload, which is characterised by low hepcidin expression [37]. However, there is no information concerning the possible compensatory effect on iron overload of the minor allele A of rs3811647. As these SNPs are found in genes for interrelated proteins that participate in one of the iron-metabolism pathways, it is suggested that the determination of these SNPs may be useful and may have clinical repercussions either in cases of iron deficiency or iron overload.

This work was focused on menstruating women because they present a high iron deficiency anaemia risk. According to WHO criteria [2], in this group of menstruating women, 10.6% were anaemic, 45.8% iron deficient and 43.7% iron sufficient women. This study replicates the associations between SNPs and serum transferrin and transferrin saturation observed previously in general population [17,18,30] and in HFE mutation carriers [38], which is remarkable, considering the size of this selected sample of menstruating women. In addition, a new finding concerning the association between H63D and serum transferrin, and the effects of the interactions between SNPs are presented.

It is well known that environmental factors, such as menstrual losses and nutrition, play an important role in the development of iron deficiency anaemia. Regarding menstruation, McLaren et al recently published a genetic association study excluding menstruating women in order to eliminate this possible confounding factor [30]. However, our results are in agreement with the association between rs3811647 and TIBC found by those authors, and in addition, an association between this SNP and transferrin saturation was found.

These data suggest that, even though there are environmental and physiological factors involved in the development of iron deficiency anaemia, there are other factors, such as genetics, that could predispose to the disease. This may influence the choice of strategies for the prevention of iron deficiency anaemia in the population. In this regard, our group carried out a placebo-controlled nutritional intervention with an iron fortified food in iron deficient women. The iron-fortified food markedly increased iron status, except in women that presented the minor allele of SNP rs3811647 [39]. These results may add new information to the area of nutrigenetics and nutrigenomics, since research on iron metabolism and gene-diet interaction is scarce.

To sum up, a large percentage of genetic variation of serum transferrin was explained by two SNPs located in the *TF *gene (rs3811647, rs1799852) and two in the *HFE *gene (C282Y, H63D) in menstruating women. In contrast to dietary intake, these SNPs are strongly associated with serum transferrin, and A carriers of rs3811647 present a reduction in iron transport to tissues, which might indicate higher iron deficiency anaemia risk. Moreover, the study of interactions of these variants shows that presence of the different alleles could have a cumulative or compensable effect in serum transferrin levels. Therefore, it is suggested that these genetic variants might potentially be used as markers of iron deficiency anaemia risk.

Further investigations in anaemic and non-anaemic subjects should be designed to increase the existing knowledge of the relationship between genetic variants and iron deficiency anaemia, and the possible modulating effect of diet and menstruation. Present results may be also useful to increase knowledge on iron overload disorders. It is also important to study the possible gene-diet interaction effects on the recovery of iron status. Finally, other studies should be carried out to explore the mechanisms by which these variants, or combination of variants as in haplotypes, affect iron metabolism at the transcriptional and postranscriptional levels and also at the functional level.

## List of abbreviations

SNP: single nucleotide polymorphism; TfR2: transferrin receptor 2; GWAS: genome-wide association study; MCV: mean corpuscular volume; BMA: bayesian model averaging; TIBC: total iron binding capacity.

## Competing interests

The authors declare that they have no competing interests.

## Authors' contributions

The authors' contributions were as follows- RBR: drafted the manuscript; RBR and AMPG: recruited the participants, collected data, performed biochemical analysis and nutrient determination; CB, AMLP and EAP participated in the genetic study design; RBR, CB, AMLP and SB performed the genotyping; ABr, ABu and JMS participated in the statistical analysis; MPV participated in the study design, contributed to the drafting, and obtained funding. All authors have read and approved the final manuscript.
